# *ARMH1 is* a novel marker associated with poor pediatric AML outcomes that affect the fatty acid synthesis and cell cycle pathways

**DOI:** 10.3389/fonc.2024.1445173

**Published:** 2024-12-05

**Authors:** Mojtaba Bakhtiari, Sean C. Jordan, Hope L. Mumme, Richa Sharma, Mala Shanmugam, Swati S. Bhasin, Manoj Bhasin

**Affiliations:** ^1^ Aflac Cancer and Blood Disorders Center, Children Healthcare of Atlanta, Atlanta, GA, United States; ^2^ Department of Biomedical Informatics, Emory University, Atlanta, GA, United States; ^3^ Department of Hematology and Medical Oncology, Emory University, Atlanta, GA, United States; ^4^ Department of Pediatrics, Emory University, Atlanta, GA, United States

**Keywords:** AML, cell cycle, fatty acid metabolic pathway, *EZH2*, *CDCA7*

## Abstract

**Introduction:**

Despite remarkable progress in Pediatric Acute Myeloid Leukemia (pAML) treatments, the relapsed disease remains difficult to treat, making it pertinent to identify novel biomarkers of prognostic/therapeutic significance.

**Material and methods:**

Bone marrow samples from 21 pAML patients were analyzed using single cell RNA sequencing, functional assays with *ARMH1* knockdown and overexpression were performed in leukemia cell lines to evaluate impact on proliferation and migration, and chemotherapy sensitivity. Mitochondrial function was assessed via Seahorse assay, *ARMH1* interacting proteins were studied using co-immunoprecipitation. Bulk RNA-seq was performed on *ARMH1*knockdown and over expressing cell lines to evaluate the pathways and networks impacted by *ARMH1*.

**Results:**

Our data shows that *ARMH1*, a novel cancer-associated gene, is highly expressed in the malignant blast cells of multiple pediatric hematologic malignancies, including AML, T/B-ALL, and T/B-MPAL. Notably, *ARMH1* expression is significantly elevated in blast cells of patients who relapsed or have a high-risk cytogenetic profile (MLL) compared to standard-risk (*RUNX1, inv (16)*). *ARMH1* expression is also significantly correlated with the pediatric leukemia stem cell score of 6 genes (LSC6) associated with poor outcomes. Perturbation of *ARMH1* (knockdown and overexpression) in leukemia cell lines significantly impacted cell proliferation and migration. The RNA-sequencing analysis on multiple *ARMH1* knockdown and overexpressing cell lines established an association with mitochondrial fatty acid synthesis and cell cycle pathways.The investigation of the mitochondrial matrix shows that pharmacological inhibition of a key enzyme in fatty acid synthesis regulation, *CPT1A*, resulted in *ARMH1* downregulation. *ARMH1* knockdown also led to a significant reduction in *CPT1A* and ATP production as well as Oxygen Consumption Rate. Our data indicates that downregulating *ARMH1* impacts cell proliferation by reducing key cell cycle regulators such as *CDCA7* and *EZH2*. Further, we also established that *ARMH1* is a key physical interactant of *EZH2*, associated with multiple cancers.

**Conclusion:**

Our findings underscore further evaluation of *ARMH1* as a potential candidate for targeted therapies and stratification of aggressive pAML to improve outcomes.

## Introduction

Pediatric Acute myeloid leukemia (pAML) accounts for about 20% of childhood leukemia (1). The prognosis and treatment of pAML remain challenging because of disease heterogeneity, high relapse rates, and therapy-associated toxicity (2). It is important to identify novel risk stratification strategies that can be used to better guide therapy decisions and improve outcomes for pAML. A deeper characterization of the pAML is essential to overcome the above-mentioned limitations. Recent studies in AML indicated that dynamic changes in the bone marrow microenvironment from the time of disease diagnosis to relapse, including alterations in the immune microenvironment, might be responsible for poor outcomes (3, 4). The therapeutics for advanced AML are undergoing a paradigm shift with the advent of immune therapy, mainly Chimeric Antigen Receptor (CAR) T-cell therapy, resulting in sustained disease responses in a subset of previously incurable patients (5). While measurable residual disease (MRD) status is critical for post-therapy patient stratification and clinical assessment, it is not infallible, with relapse occurring in some cases, even after the intense end of consolidation therapy irrespective of MRD status. It is important to identify novel risk stratification strategies that can be used to better guide therapy decisions and improve clinical outcomes for pAML patients. To achieve deep characterization of pediatric AML, Therapeutically Applicable Research to Generate Effective Treatments (TARGET) AML and other large-scale genetic profiling initiatives have generated large-scale genomics data. These initiatives indicated that AML is a genetically diverse disease having various gene mutations, insertions, and fusions associated with high risk and poor prognosis (6–8). Alongside bulk genomic data, the utilization of single-cell RNA sequencing (scRNA-seq) for profiling bone marrow microenvironment (BMM) of AML patients has revealed the molecular diversity in the blast and non-blast cells. The single-cell profiling attained better molecular characterization of AML BMM along with the identification of biomarkers/targets that can distinguish AML blast cells (AML-blasts) from non-malignant cells (non-AML cells). Single-cell profiling approaches have helped in deciphering the cellular composition of the leukemic BMM, the cellular transcriptional states associated with leukemic processes at the time of diagnosis (Dx), and the presence of residual blast cells at the end of induction (EOI) post-chemotherapy (9–13). To understand the role of tumor cells and their microenvironment in pediatric AML prognosis, we performed scRNA-seq on 21 pAML BMM samples from diagnosis (Dx), end of induction (EOI), and relapse time points (14). Analysis of matched Dx and EOI scRNA-seq data along with healthy bone marrow, hematopoietic stem cells, and TARGET AML dataset revealed a novel pAML blast-associated 7-gene signature (i.e., *CLEC11A, PRAME, AZU1, NREP, ARMH1, C1QBP, TRH*). This novel signature depicted excellent performance for pAML blast cell identification on a publicly available dataset and an independent validation cohort (14). The comparative analysis of pAML-blast cell profiles at Dx between future relapse and complete remission (CR) samples depicted overexpression of a novel Armadillo Like Helical Domain Containing 1 (*ARMH1*) gene in the relapse samples with minimal or null expression in immune and healthy stromal cells from human single-cell atlas. Higher expression of *ARMH1* was also observed in the therapy-resistant blast cells, suggesting potential involvement in the manifestation of therapeutic resistance as well as influencing outcomes. Additionally, publications from our group in characterizing T-cell acute lymphocyte leukemia (T-ALL) (15) and Mixed Phenotype leukemia (MPAL) also depicted higher expression of *ARMH1* in the leukemic cells as compared to normal cells, indicating its association with other acute leukemias. The mechanistic involvement of *ARMH1* in leukemogenesis warrants further functional and molecular studies. Correlative analysis of *ARMH1* expression with enrichment of blast cells, and leukemia risk scores depicted a significant positive association indicating *ARMH1’s* role in leukemogenesis. Further *in vitro* knockdown and overexpression of the *ARMH1* resulted in significant dysregulation in the cell proliferation and migration as well as modified sensitivity to a chemotherapy agent. The functional analysis using RNA-sequencing on *ARMH1* perturbed cells depicted a significant association with cholesterol and fatty acid biosynthesis and cell cycle (i.e., *CDCA7*, *P21*) pathways. Further, inhibition of the fatty acid pathway using a chemical inhibitor resulted in a significant reduction in *ARMH1* levels and cellular proliferation, supporting its role in disease progression. Additionally, the cellular respiration analysis using seahorse assay also depicts *ARMH1’s* impact on mitochondrial cellular respiration. Therefore, this study provides the first evidence about the molecular mechanism of *ARMH1* in leukemogenesis that can be explored in future clinical trials to develop novel therapies.

### *ARMH1* expression is associated with a higher disease burden in pediatric leukemias

**Figure 1:**
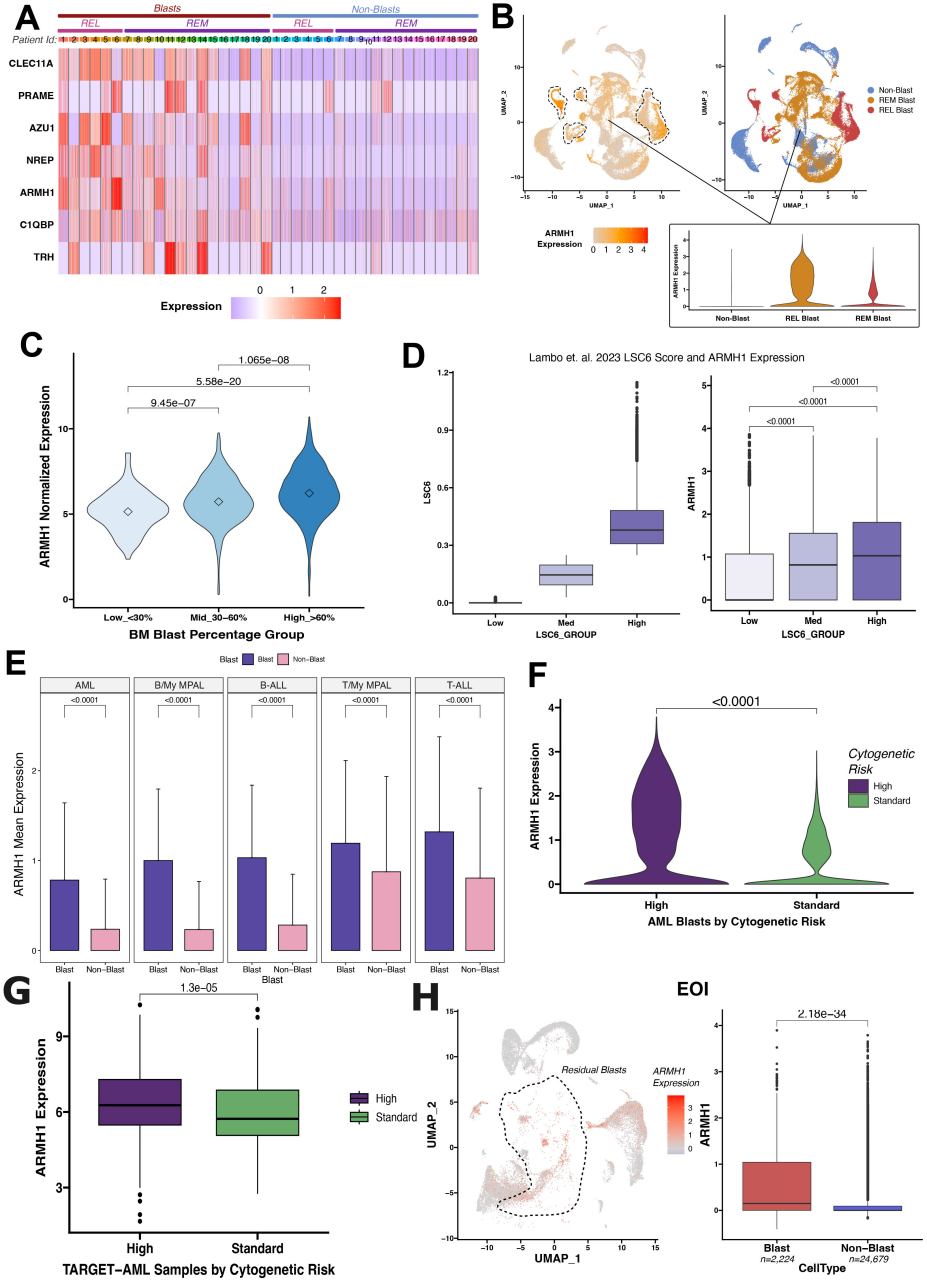
Differential *ARMH1* expression in leukemic blasts revealed by scRNA-seq analysis. **(A)** Heatmap showing expression of the genes from the seven gene signatures across blast and non-blast cells in 20 AML samples collected at disease diagnosis. Patient IDs are represented by numbers along columns and are grouped into REL (relapse) and REM (remission) groups based on their outcome. Blue and red colors represent low and high expression, respectively. **(B)** UMAPs showing *ARMH1* expression in scRNA-seq data of AML samples at diagnosis (left) with low to high expression colored light to intense orange colors. Blast clusters are lassoed on the feature plot. The UMAP plot (right) shows cellular grouping with non-blast cells shown in blue and blast cells from remission (REM) and relapse (REL) in orange and red colors, respectively. An inset violin plot shows the *ARMH1* expression in non-blast immune microenvironment cells, remission, and relapse blast cells. **(C)** Violin plot with *ARMH1* expression in the bulk RNA-seq TARGET-AML samples stratified based on clinical blast percentages. Samples are grouped based on diagnosis blast percentages [Low (<30%), Medium (30%-60%), High (>60%)]. Points represent median values per group. **(D)** AML blast cells from (16) study are grouped by pLSC6 score (left). *ARMH1* expression is plotted for each pLSC6 group (low, mid, high) (right). **(E)** Mean and standard deviation of *ARMH1* expression in blast (purple) and non-blast (pink) cells across different pediatric acute leukemias: AML (acute myeloid leukemia), B-MPAL (B-cell/Myeloid mixed phenotype acute leukemia), B-ALL (B-cell acute lymphoid leukemia), T-MPAL (T-cell/Myeloid MPAL), and T-ALL (T-cell ALL). **(F)** Violin plot of *ARMH1* expression in diagnosis AML samples categorized by cytogenetic risk. High risk (purple) represents MLL patients, and standard risk (green) represents RUNX1 and inv (16) patients. **(G)** *ARMH1* expression in TARGET-AML BM samples collected at disease diagnosis from patients with different cytogenetic risk categories [high risk=MLL, standard risk=*RUNX1, inv (16)*]. The significance of differences in expression levels throughout the analysis was determined using the Wilcoxon Rank-Sum test. The Bonferroni method was used to adjust p-values for multiple comparisons. If the p-value is below 0.0001, it is indicated on the plots. **(H)** UMAP with *ARMH1* expression in the residual blast and non-blast immune microenvironment cells identified from comparison of DX and End of Induction (EOI) samples. Blast clusters are lassoed on the UMAP. The boxplot shows significantly higher expression of *ARMH1* in residual blast cells at EOI compared to non-blast immune cells.

Our group conducted a single-cell RNA sequencing (scRNA-seq) analysis of pediatric AML BMM samples for in-depth characterization of leukemic blasts and other tumor microenvironment cells (14). A unique AML-blast associated 7-gene signature was identified by analyzing integrated scRNA-seq data from matched Dx and EOI AML BM samples and TARGET AML bulk dataset by implementing a supervised machine learning technique, support vector machine (SVM) approach. The seven gene signature achieved excellent performance on training and independent validation sets in distinguishing AML blast cells from the immune microenvironment cells (AUC >0.78) (14). The signature also depicted elevated expression in malignant blast cells as compared to other non-blast (microenvironment cells) profiled in the 20 patient samples by the single cell assay (**Figure 1A**). Further analysis of AML samples at diagnosis revealed that *ARMH1* expression not only blast-specific but the expression was also elevated in the blast cells from patients that relapsed (marked with dotted lasso) in comparison to blast cells from patients who achieved Clinical Remission (CR) (**Figure 1B**). In our previous study, *ARMH1* expression was observed to be significantly higher in scRNA-seq data of AML Dx blast cells compared to EOI non-blast cells (*P<0.001*) (14). *ARMH1*, also known as *C1orf228*, has not been previously associated with AML or other pediatric cancers. *ARMH1* encodes for Armadillo-like helical domain containing protein1 with unknown function and association with diseases. To investigate its involvement in AML, we performed further validation on large-scale TARGET AML RNA-seq data. *ARMH1* depicted an association with clinical blast percentage, with expression slightly increasing as blast percentages increase from <30% to >60% (**Figure 1C**). This finding provides evidence of *ARMH1* blast cells associated expression. Additionally, expression of *ARMH1* in AML malignant cells from an external pediatric AML study (16) was correlated with the enrichment of a leukemia stem cell score of 6 genes (LSC6) that predicts AML outcome reliably with high LSC6 scores associated with poor outcome (17). When malignant cells are stratified by their LSC6 scores into three groups, *ARMH1* expression incrementally increases from low to medium to high LSC6 groups (*P<0.0001*). The malignant AML cells from this study with the highest LSC6 scores also had the highest *ARMH1* expression (**Figure 1D**). Besides AML, our group also performed in-depth characterization of other pediatric leukemias, Acute lymphocytic leukemia (T-ALL, B-ALL) and mixed phenotypic acute leukemia (T- MPAL, B-MPAL) (18). *ARMH1* depicted significantly higher expression in T-ALL (*P<0.0001*), B-ALL (*P<0.0001*), T-MPAL (*P<0.0001*), B-MPAL (*P<0.0001*), blast cells as compared to normal non-blast cells (**Figure 1E**). This pattern of blast cell restrictive expression of *ARMH1* indicates its potential association with leukemogenesis. Moreover, the investigation of levels of *ARMH1* expression in samples from three patients with a high-risk cytogenetic profile (MLL) and six patients with a standard risk cytogenetic categorization [*RUNX1*, *inv (16)*] revealed that *ARMH1* expression was significantly higher (*P<0.0001*) in high-risk (MLL) compared to standard-risk [*RUNX1*, *inv (16)*] group (**Figure 1F**). This is further validated in TARGET-AML BM diagnosis samples showing significantly (*P=1.3e-05*) higher *ARMH1* expression in the high-risk cytogenetic subtypes (i.e., MLL) compared to standard-risk [e.g., *RUNX1, inv (16)*] (**Figure 1G**). Utilizing temporal AML samples collected at Dx, EOI, and Relapse timepoints of the same patients, we assessed *ARMH1* expression in blast cells across disease stages. We observed *ARMH1* expression was mostly localized to blast cells across time points, in blasts at diagnosis, as well as residual blasts at EOI (*P=2.18e-34*). (**Figure 1H**). A survival analysis of *ARMH1* expression in pediatric AML data from TARGET depicted a significant association between *ARMH1* (*P=0.00199*) overexpression and poor overall survival. These correlative analysis results provide substantial evidence for *ARMH1* association with high-risk or aggressive pediatric acute leukemias, specifically AML. These lay the foundation to evaluate the functional impacts of *ARMH1* through perturbation studies described in the following sections of the manuscript.

### Knockdown and overexpression of *ARMH1* modulate AML cell proliferation, migration, and susceptibility to chemotherapy

**Figure 2:**
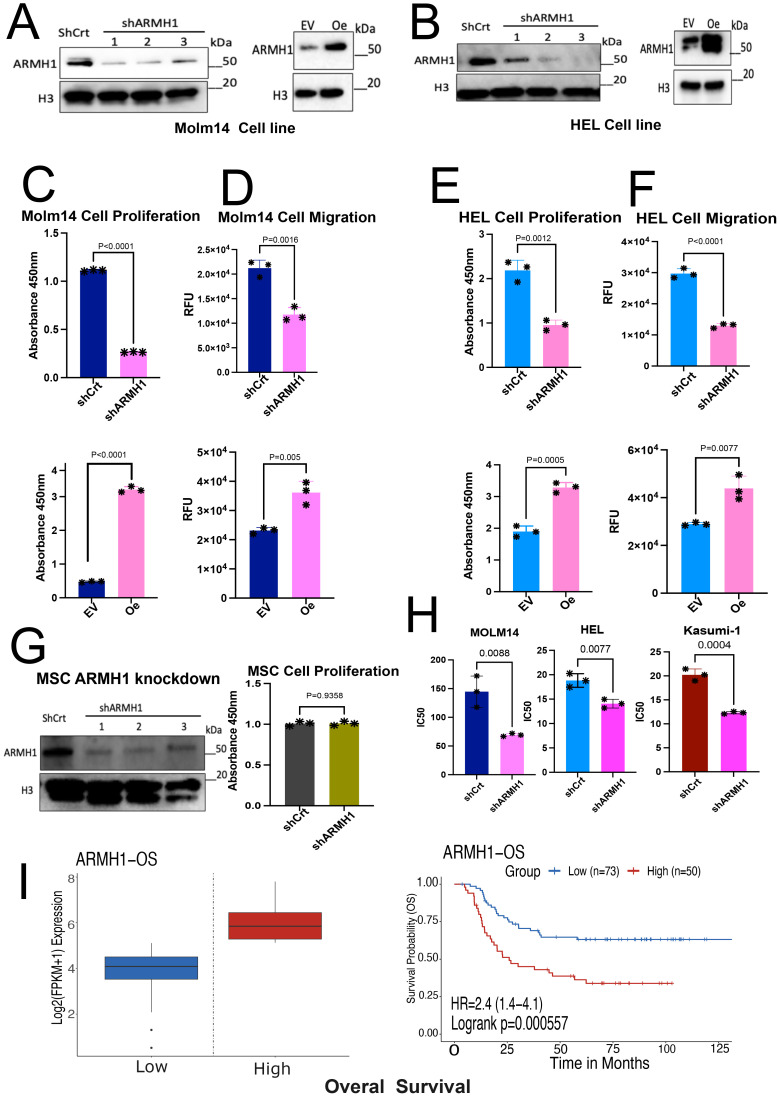
Knockdown and overexpression of *ARMH1* modulate AML cell proliferation and migration and better susceptibility to chemotherapy. **(A)** Left panel: Western blot of MOLM 14 cell lysates probed with *ARMH1* antibody and total H3 antibody as loading control (3 independent replicates) with lentiviral transduced *ARMH1* knockdown (*shARMH1)* and shControl (shCrt). Right panel: *ARMH1* overexpressed (Oe) using lentiviral transduced in MOLM14 cells with ORF (pLX-317) or Empty Vector (EV). **(B)** Left panel: Western blot of HEL92.1.7 cell lysates probed with *ARMH1* antibody and total H3 antibody as loading control in lentiviral transduced *shARMH1* and shCrt cells. Right panel: *ARMH1* Oe in lentiviral transduced ORF (pLX-317), and EV. **(C)** Bar plots showing cell proliferation measured by CCK-8 in MOLM14 *shARMH1* cells along with shCrt in the top panel, and MOLM14 cells with *ARMH1* EV along with *ARMH1* Oe in the lower panel. Statistical analysis: Unpaired student’s t-test (n=3), data are means (absorbance in 450nm) +/-SEM of independent biological replicates (n=3). **(D)** Bar plots showing the migration assay results that were conducted by using the fluorometric format in MOLM14 cell lines with *shARMH1*, shCrt along with EV and *ARMH1* Oe, the results of *shARMH1* and *ARMH1* Oe are shown in the top and bottom panels respectively. Statistical analysis: Unpaired student’s t-test (n=3), data are means of (Relative Florescence Unit (RFU) +/-SEM of independent biological replicates. **(E)** Bar plots showing cell proliferation measured by CCK-8 of HEL92.1.7 with *shARMH1* cells along with shCrt in the top panel, and HEL92.1.7 cells with EV along with *ARMH1* Oe HEL 92.1.7 cells in the lower panel. Statistical analysis: Unpaired student’s t-test (n=3), data are means (absorbance in 450nm) +/-SEM of independent biological replicates. **(F)** Bar plot showing the migration assay results of HEL92.1.7 cell line with *shARMH1*, shCrt along with EV, and *ARMH1* Oe. The HEL92.1.7 migration assay results for *shARMH1* and *ARMH1* Oe are shown in the top and bottom panels, respectively. Statistical analysis: Unpaired student’s t-test (n=3), data are means of (Relative Florescence Unit (RFU) +/-SEM of independent biological replicates. **(G)** Left panel: Western blot of Mesenchymal Stromal Cell Line (MSC) lysate probed with *ARMH1* antibody and total H3 antibody as loading control with lentiviral transduced shRNA for targeting *ARMH1* and shCrt (3 independent replicates). The lower bands in H3 are non-specific bands. Right panel: Bar chart of cell proliferation measured by CCK-8 in MSC *shARMH1*, and shCrt. Statistical analysis: Unpaired student’s t-test (n=3), data are means (absorbance in 450nm) +/-SEM of independent biological replicates. **(H)** The bar graph shows individual IC_50_ values for each of the three independent biological replicates on the Y-axis. IC_50_ (Cytarabine treatment, nM) reported for *shARMH1* and shCrt cells that were exposed to increasing concentration of Cytarabine in MOLM14 (*p=0.0088*), HEL92.1.7 (*p=0.0077*) and Kasumi-1 (*p=0.0004*). The *p-value* was calculated using a two-tailed Student’s t-test on graph pad prism 10. **(I)** Kaplan-Meier curve shows the overall survival in pAML patients of the TARGET dataset, grouped by *ARMH1* expression. High expression of *ARMH1* is associated with poor outcomes in this cohort (HR=2.4 (95% CI=1.4-4.1) log-rank *p=0.000557*). The Survival Genie platform (https://bhasinlab.bmi.emory.edu/SurvivalGenie2/home) was used to perform survival analysis. The samples were grouped into “low” and “high” groups based on an optimal threshold calculated using the cutP algorithm. Using these groupings, we calculate the survival probability and generate Kaplan Meier curves with corresponding log-rank p-value, which represents the significance of the difference between the curves for “high” and “low” expression groups. A Cox proportional hazard ratio (HR) is also calculated between the high and low samples; an HR>1 represents poor survival, whereas an HR<1 represents an association with better survival.

Following promising findings regarding the increased expression of *ARMH1* (**Figure 1**) in malignant and therapy-resistance aggressive AML cells, we initiated functional studies to delve into the role of *ARMH1* in leukemogenesis. To evaluate the functional impact of *ARMH1*, we modulated the expression of *ARMH1* by knockdown and overexpression and measured the effect on cellular proliferation and migration. We achieved *ARMH1* knockdown (*shARMH1*) vs. wild type of *ARMH1* (shCrt) and overexpression (*ARMH1* Oe) vs wild type of *ARMH1* (empty vector=EV) in three different AML cell lines, MOLM14 (n=3), HEL92.1.7 (n=3), and only knockdown in Kasumi-1 (n=3). The knockdown and overexpression were confirmed by western blot in MOLM14 (**Figure 2A**), HEL92.1.7 (**Figure 2B**), and Kasumi-1 cell lines (**Supplementary Figure 1A**). We next performed cellular proliferation, migration, and dose-response assays. The cellular proliferation analysis revealed a substantial reduction in cell proliferation upon knockdown of *ARMH1* in MOLM14 (*P<0.0001)*, HEL92.1.7 (*P=0.0012)*, whereas a significant increase in cell proliferation upon *ARMH1* overexpression in MOLM14 (*P<0.0001*), HEL92.1.7 (*P=0.0005*) (**Figures 2C, E**) was observed. Likewise, we observed a significant decrease in cell proliferation in *ARMH1* knockdown in Kasumi-1 (*P<0.0001*) (**Supplementary Figure 1B**) cell lines. Analysis of cell migration showed that the knockdown of *ARMH1* led to a significant decrease in cell migration in MOLM14 (*P=0.0016*) and HEL92.1.7 (*P<0.0001*), while overexpression significantly increased cell migration in MOLM14 (*P=0.005*) and HEL92.1.7 (*P=0.0077*) cell lines (**Figures 2D, F**). A significant decrease in migration was also noted upon *ARMH1* knockdown in Kasumi-1 (*P<0.0001*) (**Supplementary Figure 1C**). To verify the impact of *ARMH1* on propagation in normal non-leukemic cells, we also performed the knockdown of *ARMH1* in normal human cell lines, HEK293T, and Mesenchymal stromal cells (MSC) cells. After the knockdown of *ARMH1*, no significant alteration in cell proliferation was observed in MSCs (*P=0.9358)* (**Figure 2G**) and HEK293T (*P=0.3925)* (**Figure 1D**). Additionally, we investigated *ARMH1’*s potential role in altering the therapeutic index of traditional chemotherapy (i.e., Cytarabine) for pAML. We observed an improvement in sensitivity to Cytarabine in *ARMH1* knockdown relative to wild-type in MOLM14 (n=3, *p=0.0088*), HEL92.1.7 shCrt vs *shARMH1* (n=3) (*p=0.0077*), and Kasumi-1 shCrt vs *shARMH1* (n=3) (*p=0.0004*) (**Figure 2H**). The survival analysis on *ARMH1* expression in pAML data from the TARGET dataset performed using the Survival Genie tool (n=144) depicted a significant association between *ARMH1* (HR=2.4, *P=0.00199*) overexpression and poor overall survival (19) (**Figure 2I**). These results underscore the involvement of *ARMH1* in cancer cell proliferation, migration, and on mediating resistance to therapeutic regimens. To further understand the molecular mechanism of *ARMH1*, we performed RNA-seq on *ARMH1* perturbed cells to measure genome-level transcriptome dysregulations.

### Bulk RNA sequencing analysis of *ARMH1* perturbed cells for in-depth characterization of gene and pathway level alterations

**Figure 3:**
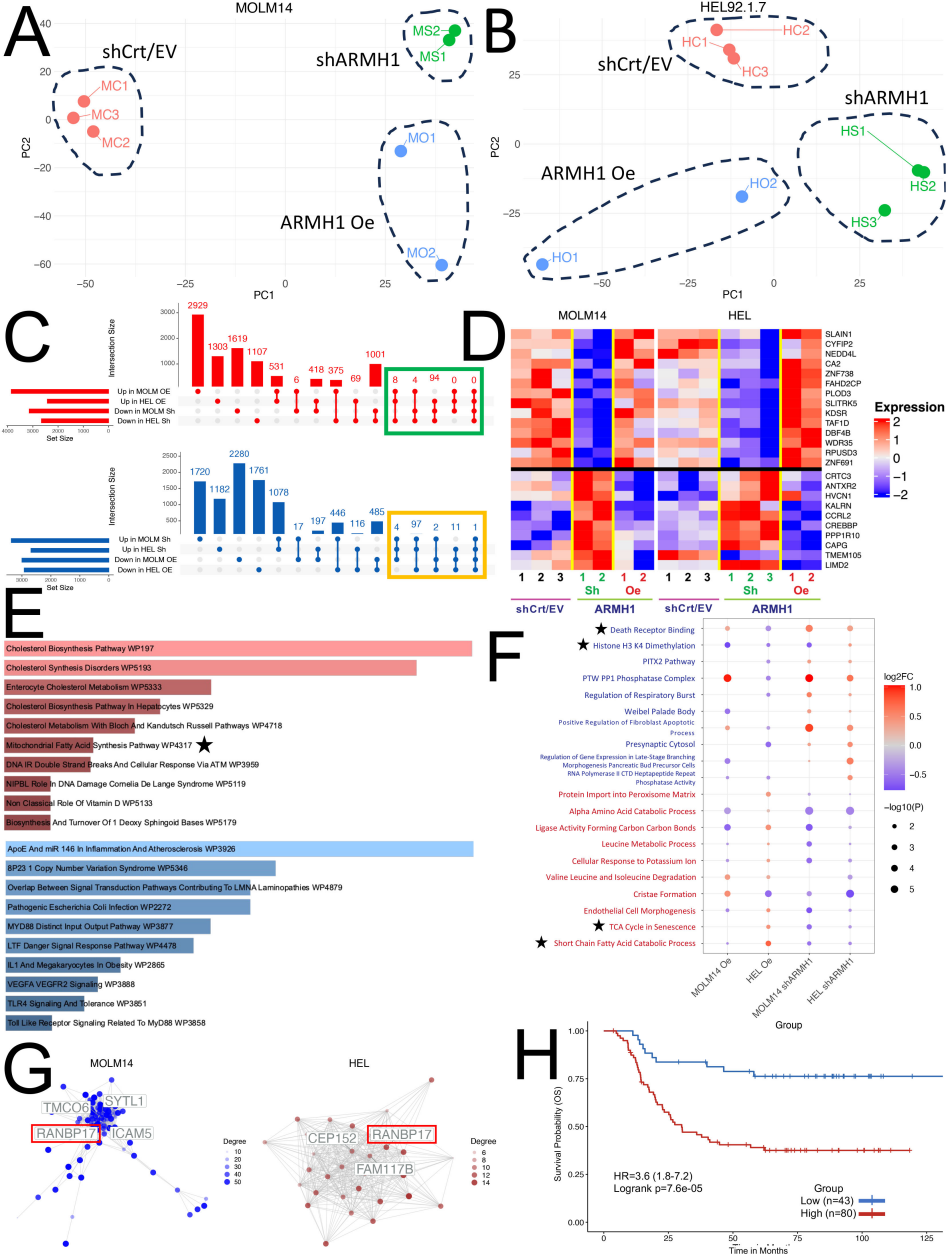
Transcriptome profiling of *ARMH1* perturbed cells for exploration of gene and pathway alterations. **(A)** PCA plot with first two principal components derived from the normalized gene expression matrix using PCA for the seven samples obtained from the MOLM14 cell line. Each dot represents a sample, and distinct separation among treatment groups (*ARMH1* Oe, shCrt/EV, and *shARMH1*) is observed. **(B)** Similar to **(A)**, the PCA plot illustrates the first two principal components for the eight samples collected from the HEL cell line after the PCA transformation of the normalized gene expression matrix. Notable separation among treatment groups is evident. **(C)** UpSet plot displaying intersecting genes in MOLM14 and HEL92.1.7 cell lines that are perturbed by *ARMH1* Oe and *shARMH1*. The top plot (red) shows co-expressing genes significantly upregulated in *ARMH1* Oe and downregulated in *shARMH1*, and the bottom plot (blue) shows counter-regulated genes significantly downregulated in *ARMH1* Oe and upregulated in *shARMH1* at *p < 0.05*. Genes selected for downstream analyses (significant at *P < 0.05* in at least three comparisons and consistent fold change) are highlighted with green and orange boxes. **(D)** Heatmap displaying relative expression values for selected *ARMH* co-expressed (genes from the green box in **(C)** (top) and counter-regulated (genes from the orange box in **(C)** (bottom). All genes were significant at *p < 0.05* in at least three out of four comparisons. Gene expression is shown in pseudocolor with red denoting increase and blue decrease in gene expression. The rows and columns represent the genes and samples, respectively. **(E)** The top 10 pathways upregulated in *ARMH1* co-expressed gene (red), as well as the top 10 pathways downregulated in *ARMH1* counter-regulated genes. **(F)** Dot plot of the top 10 gene sets following a similar or co-expression pattern as *ARMH1* (text in red) and gene sets following the opposite or counter-expression pattern as *ARMH1* (text in blue). Gene sets are significant in at least three of the four contrasts at p < 0.05 and are ordered by the average of the absolute value log2FCs from the four comparisons listed on the x-axis. **(G)** Gene co-expression networks generated from *ARMH1* co-expressed genes. Hub genes are labeled, with the gene common between both cell lines with the highest degree of connections. **(H)** Survival association analysis of *ARMH1* co-expressed genes using pAML data from the TARGET initiative, with groups stratified based on enrichment score for *ARMH1* co-expressed genes. Patients with higher enrichment scores for *ARMH1* co-expressed genes have poor survival (HR=3.6 (95% CI=1.8-7.2) log-rank *p=0.000076*). The symbol ★ are notable pathways selected for further analysis.

To characterize gene expression level alterations in *ARMH1* perturbed cells and explore the affected pathways, bulk RNA sequencing was performed. Unsupervised Principal Component Analysis illustrates distinct separations among the three treatment groups (*ARMH1* Oe, shCrt/EV, and *shARMH1*) for MOLM14 (**Figure 3A**) and HEL92.1.7 cell lines indicating distinct transcriptome profiles of groups (**Figure 3B**). The differential expression analysis on MOLM14 cell lines identified 2,924 and 3,276 significantly (Adj. *P < 0.01*) differentially expressed (upregulated as well as downregulated) genes, respectively upon *ARMH1* knockdown and overexpression. Similar analysis on *ARMH1* knockdown and over-expressing HEL92.1.7 cell lines identified 1,777 and 1,184 significantly (Adj. *P < 0.01*) differentially expressed genes, respectively. To identify genes that followed the same expression patterns as *ARMH1* (i.e. increased with *ARMH1* overexpression and decreased with *ARMH1* knockdown), we performed a comparative analysis of differentially expressed genes using UpSet plots (20) (**Figure 3C**, top plot in red) and those that followed the opposite expression pattern as *ARMH1* (i.e. decreased with *ARMH1* overexpression and increased with *ARMH1* knockdown; the bottom plot in blue). The analysis identified 106 genes that were significantly (*P < 0.05*) upregulated with *ARMH1* overexpression and downregulated with *ARMH1* knockdown in at least three of the four contrasts. Conversely, there was one gene, *LIMD2*, that was significantly downregulated with *ARMH1* overexpression and upregulated with *shARMH1* in all four contrasts (*P-value*: MOLM14 *ARMH1* Oe = 0.0271, MOLM14 *shARMH1 = 0.0196*, HEL *ARMH1* Oe= *0.0016*, HEL *shARMH1 = 0.0001*), and 114 other genes that were significant in at least three of the four contrasts at *P < 0.05*. Therefore, we considered 106 genes that followed the same expression patterns as *ARMH1* and 115 genes that followed the opposite expression patterns as an *ARMH1* perturbed core signature. The expression levels of selected genes from this core signature were visualized in the heatmap depicting very concordant patterns across *ARMH1* perturbed (i.e., knockdown, overexpression) and control cell lines (MOLM14, HEL92.1.7) (**Figure 3D**). Among the top genes co-regulated with *ARMH1* include *SLAIN1, CYFIP2, NEDD4L, CA2, ZNF738, FAHD2CP, PLOD3, SLITRK5, KDSR, TAF1D, DBF4B, WDR35, RPUSD3* and *ZNF691*. Several of these genes were studied by other teams, such as *SLAIN1* (21) (previously reported to contribute to cancer cell proliferation and therapy resistance) as well as *CYFIP2* (22) and *CA2* (23) (currently being explored as prognostic biomarkers). Pathway analysis using the 106 genes (genes boxed in green in **Figure 3C**; co-regulated with *ARMH1*) depicts the significant enrichment of pathways related to cholesterol biosynthesis (*P = 0.0023*) and mitochondrial fatty acid synthesis (*P = 0.0288*). For the second group (genes boxed in yellow in **Figure 3C**; showing opposite trends (counter-regulated) to *ARMH1* protein expression), the pathways relating to the overall regulation of inflammation (*P = 0.0007*) as well as the angiogenesis pathway through *VEGFA* and *VEGFR2* (*P = 0.0084*) were significantly enriched (**Figure 3E**). Further gene set enrichment analysis on the normalized expression values of co-regulated genes depicted upregulation of TCA cycle in senescence (*P-value: MOLM14 shARMH1 = 0.0024, HEL ARMH1* Oe *= 0.0118, HEL shARMH1 = 0.0291*) and short-chain fatty acid catabolic processes (*P-value: MOLM14 ARMH1* Oe *= 0.0450, MOLM14 shARMH1 = 0.0111, HEL ARMH1* Oe *= 0.0014, HEL shARMH1 = 0.0254*) while analysis on the genes with an opposite expression pattern depicted perturbation of death receptor signaling (*P-value: MOLM14 ARMH1* Oe *= 0.0050, MOLM14 shARMH1 = 3.04e-5, HEL ARMH1* Oe *= 0.0013, HEL shARMH1 = 0.0007*) and Histone H3 K4 Dimethylation (*P-value: MOLM14 ARMH1* Oe *= 0.0010, MOLM14 shARMH1 = 0.0029, HEL ARMH1* Oe *= 0.0162, HEL shARMH1 = 0.0282*) (**Figure 3F**). Further co-expression network analysis on the co-regulated genes revealed one gene, RAN binding protein 17 (*RANBP17*) that was identified as a central hub gene in both the MOLM14 and HEL co-expression Networks (**Figure 3G**). *RANBP17* is a member of the importin-beta superfamily of nuclear transport receptors. The expression of *RANBP17* has been implicated in the progression of many cancers (24, 25), and it was also reported as a potential prognostic biomarker for glioblastoma (26). Additionally, *RANBP17* is reported to be a member of the *ARMH1* superfamily, according to the Hugo Gene Nomenclature Committee, suggesting that it could be closely related to *ARMH1* in both structure and function (27). To further investigate the overall impact of the *ARMH1* co-expressed genes on AML outcomes, we performed a survival analysis using the TARGET-AML cohort. The survival analysis showed that higher expression levels of *ARMH1* co-expressed genes (n=88 genes with valid gene symbols out of the 106 co-expressed genes) were significantly associated (HR=3.6, *P=0.0003*) with poorer overall survival (**Figure 3H**). In summary, the bulk RNA-seq data suggests *ARMH1* is likely a key component of the cellular metabolic process, particularly in the cholesterol and fatty acid synthesis pathways that occur in the mitochondria. Next, we investigated the role of *ARMH1* in fatty acid synthesis in mitochondria as well as in cell cycle regulation.

### *ARMH1* shows an association with mitochondrial fatty acid synthesis and cell cycle regulation

**Figure 4:**
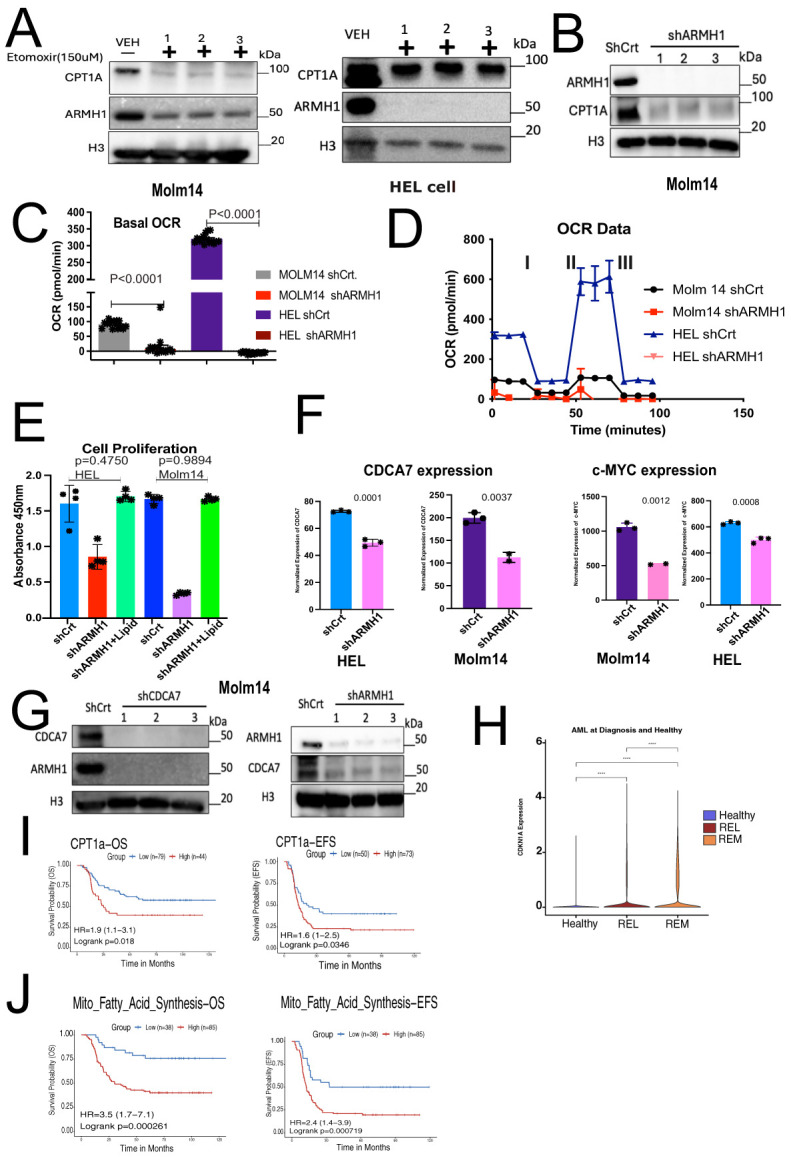
*ARMH1* shows potential association with mitochondrial fatty acid synthesis and Cell cycle regulation. **(A)** Left panel: western blot shows the inhibition of *CPT-1A* and *ARMH1* by Etomoxir (150uM) and DMSO control treatment of the MOLM14 cell line (3 independent replicates). Right panel: western blot shows the inhibition of *CPT-1A* and *ARMH1* by Etomoxir (150uM) and DMSO control treatment of the HEL92.1.7 cell line (3 independent replicates). **(B)** Western blot of MOLM14 cell lysates probed with *ARMH1*, *CPT1A* antibody, and total H3 antibody as loading control with lentiviral transduced *shARMH1* or shCrt (3 independent replicates). The *CPT-1A* expression is significantly lower in cells with *ARMH1* knockdown cells. **(C)** Bar plot showing an Oxygen Consumption Rate (OCR) in *shARMH1* and shCrt MOLM14 and HEL92.1.7 cells. Statistical analysis: Unpaired student’s t-test (n=15), data are means of [OCR (pmol/min)] +/-SEM of independent biological replicates. **(D)** Respiration stress test indicates the basal respiration and reflects the energy requirements under conditions, IA=Oligomycin (2.5uM), IIB=FCCP (0.5uM), and IIIC=Antimycin (2uM) and Rotenone (2uM). **(E)** Bar plot showing cell proliferation assay in *shARMH1* and shCrt MOLM14 and HEL92.1.7 cells, the cell culture media supplemented with exogenous lipid mix (Lipid Mixture 1, Cat#10288, Sigma-Aldrich). The proliferation was measured at 72 hours. Statistical analysis: Unpaired student’s t-test, data are means (absorbance in 450nm) +/-SEM of independent biological replicates (n=4). **(F)** Left panel: Bar plot of normalized expression of *CDCA7* and Right panel: *c-MYC* in *shARMH1* and shCrt MOLM14 and HEL92.1.7 cells. Statistical analysis: Unpaired student’s t-test, data are normalized expression+/-SEM of *c-MYC* and *CDCA7* from independent biological replicates (HEL92.1.7 Control/Knockdown, MOLM14 Control: n=3, HEL92.1.7 Overexpression, MOLM14 Knockdown/Overexpression: n=2). **(G)** Left Panel: Western blot of cell lysate probed with *ARMH1*, *CDCA7*, and total H3 as loading control with lentiviral transduced *shCDCA7* and shCrt. (3 independent replicates). Right panel: Western blot of MOLM14 cell lysates probed with *ARMH1* and *CDCA7* antibody and total H3 antibody as loading control with lentiviral transduced *shARMH1* and shCrt (3 independent replicates). **(H)** Log-normalized expression of *CDKN1A* in pAML bone marrow diagnosis (n=20) and healthy (n=4) samples. AML samples are grouped into REL (relapse), and REM (remission) based on future outcomes. Wilcoxon Rank-Sum test was used to compare expressions between groups, and p-values were reported (*p<0.0001*). **(I)** Upper panel: Kaplan-Meier curve shows overall survival (left) and event-free survival (EFS) (right) of pAML samples from the TARGET initiative by stratifying samples into high and low groups based on *CPT1A* expression. Lower panel: Kaplan-Meier curve shows overall survival (left) and event-free survival (EFS) (right) of pAML samples from the TARGET initiative by stratifying samples into high and low groups based on enrichment score for mitochondrial fatty acid synthesis gene set obtained from MsigDB via msigdbr (v7.5.1) from the Broad Institute. The Survival Genie platform (https://bhasinlab.bmi.emory.edu/SurvivalGenie2/home) was used to perform survival analysis. The samples were grouped into “low” and “high” groups based on an optimal threshold calculated with the cutP method. Using these groupings, we calculate the survival probability and generate Kaplan Meier curves with corresponding log-rank p-value, which represents the significance of the difference between the curves for “high” and “low” expression groups. A Cox proportional hazard ratio (HR) is calculated between the high and low samples, an HR>1 represents poor survival, whereas an HR<1 represents an association with better survival. The asterisk (-) means 0 concentration of drug.

AML cells exhibit an abnormal metabolic profile, which includes a higher need for Oxidative Phosphorylation and Fatty Acid Metabolism for survival and therapeutic resistant phenotype (28). Our RNA-seq analysis on *ARMH1* knockdown and overexpression cells revealed that the mitochondrial fatty acid synthesis pathway was significantly (*P=0.02875*) dysregulated (**Figure 3F**) and provided clues for an underlying mechanism for *ARMH1* association with leukemogenesis. To investigate *ARMH1* association with Oxidative phosphorylation and ATP production, we first focused on studying the fatty acid Oxidative cycle by targeting a key enzyme associated with transferring long-chain fatty acids into mitochondria, which is located in the outer layer of the mitochondria membrane (29), Carnitine palmitoyl transferase 1A (*CPT1A*). By pharmaceutical inhibition of *CPT1A*, using Etomoxir (150uM), we observed that *ARMH1* expression was decreased by inhibiting *CPT1A* in MOLM14 and HEL92.1.7 cell lines (**Figure 4A**). We also observed a reduction in the expression of *CPT1A* following the knockdown of *ARMH1* in MOLM14 cell lines indicating potential co-regulations of *ARMH1* and *CPT1A* (**Figure 4B**). In addition, we observed that *CPT1A* has significant co-expression with *ARMH1* in AML samples from TARGET cohort (*r=0.18, P=3.5e-14*) (**Supplementary Figure 2C**). *CPT1A* has a significant role in mitochondrial fatty acid synthesis, and its expression is associated with increased cell proliferation and suppression of apoptosis in various cancers, including leukemia (30–32). Overexpression of *CPT1A* has been consistently linked to adverse outcomes in a variety of cancers, including AML (30, 33). Targeting *CPT1A* in leukemia cell lines leads to a decrease in cancer cell proliferation (34) and a reduction in apoptosis (35). To substantiate these findings, we performed the Seahorse assay to measure the Oxygen Consumption Rate (OCR) in both MOLM14 and HEL92.1.7 *ARMH1* knockdown and control wild-type cells. The data demonstrated a notable reduction in OCR in *ARMH1* knockdown in HEL 92.1.7 (*P<0.0001)* and MOLM14 (*P<0.0001*) cells compared to respective controls (**Figures 4C, D**). We also investigated the involvement of *ARMH1* in the regulation of mitochondrial fatty acid synthesis by conducting a rescue study. We supplemented the cell culture media with an exogenous lipid mixture and conducted a cell proliferation assay in *ARMH1* knockdown compared to wild-type conditions. The supplementation of lipid mixture restored cell proliferation of *ARMH1* knockdown in MOLM14 and HEL92.1.7 cells to a level similar to control wild-type cells. This data indicated that the lipid mixture compensates for the reduction in proliferation due to *ARMH1* knockdown (**Figure 4E**). In summary, these findings provide preliminary evidence of *ARMH1* involvement in mitochondrial fatty acid synthesis in leukemia cells.

In addition to impacting mitochondrial fatty acid synthesis, RNA-sequencing data also shows that *ARMH1* is associated with the dysregulation of the cell cycle by affecting the expression of key cell cycle regulators such as Cell Division Cycle Associated 7 (*CDCA7*) and Cyclin-dependent kinase inhibitor 1A (*CDKN1A or P21*) genes. *CDCA7* has been previously reported to play a significant role in cancer progression in diverse types of malignancies (36–41). *CDCA7* shows periodic expression in the cell cycle, peaking between G1/S phases (42). Studies have also demonstrated a connection between *CDCA7* and *c-MYC*, a potent cancer oncogene (43). In our RNA sequencing data, we identified that expression of *CDCA7* and *c-MYC* significantly decreased in *ARMH1* knockdown cell lines for *CDCA7*, MOLM14 wild type compared to *shARMH1* (*P=0.0035*), and HEL92.1.7 wild type compared to *shARMH1* (*P=0.0002*) and for *c-MYC* MOLM14 wild type compared to *shRAMH1* (*P=0.0005*) and HEL92.1.7 wild type compared to *shARMH1* (*P=0.0002*) (**Figure 4F**). Further, correlative analysis of using AML data from TARGET cohort revealed *ARMH1* expression has a significant correlation with *CDCA7* (*r=0.29, P<2.2e-16*) and *c-MYC* (*r=0.2, P=4.6e-16*) which suggests intersection between their regulatory pathways in leukemia cells (**Supplementary Figure 2C**). Through *in vitro* assays, we observed a co-expression between *ARMH1* and *CDCA7*. Knockdown of *ARMH1* led to a remarkable reduction in *CDCA7* expression. Conversely, after the knockdown of *CDCA7*, the expression of *ARMH1* also exhibited a similar reduction in MOLM14 (**Figure 4G**). A similar co-expression pattern was observed between *ARMH1* and *CDCA7* in HEL92.1.7, underscoring the co-regulation or interaction (**Supplementary Figure 2D**). In addition, we also investigated the association between *ARMH1* and *P21*, which function as a suppressor of the cell cycle, thereby inhibiting cell growth (44, 45). Our Bulk RNA-seq analysis depicted an upregulation of *P21* in *ARMH1* knockdown in MOLM14 (*P=0.0106)*, and in HEL 92.1.7 (*P=0.0320*) cell lines as compared to wild-type controls (**Supplementary Figure 2E**). The analysis of our single-cell data (14), including bone marrow samples of healthy control and patients at diagnosis who went on to experience relapse or remission, reveals significant overexpression of *P21* in remission patients compared to relapse (*P<0.0001*) (**Figure 4H**). Further, survival analysis on *CPT1A* and mitochondrial fatty acid pathway (MSigDB, GO:0019395) expression in pediatric AML data from the TARGET initiative depicted a significant association between *CPT1A* (OS *P=0.00249, EFS P=0.047*) and mitochondrial fatty acid synthesis (OS *P=0.000837, EFS P=0.00085*) overexpression with poor overall and event-free survival in TARGET-AML cohort (**Figure 4I**).

Collectively, these findings indicate *ARMH1* is involved in the modulation of the mitochondrial fatty acid synthesis pathway in pediatric AML. Further, in the absence of *ARMH1*, the cell cycle is arrested by upregulation of the checkpoint inhibitor, *P21*, and downregulation of the expression of *CDCA7*.

### *ARMH1* is a novel interactant of *EZH2* in leukemia

**Figure 5:**
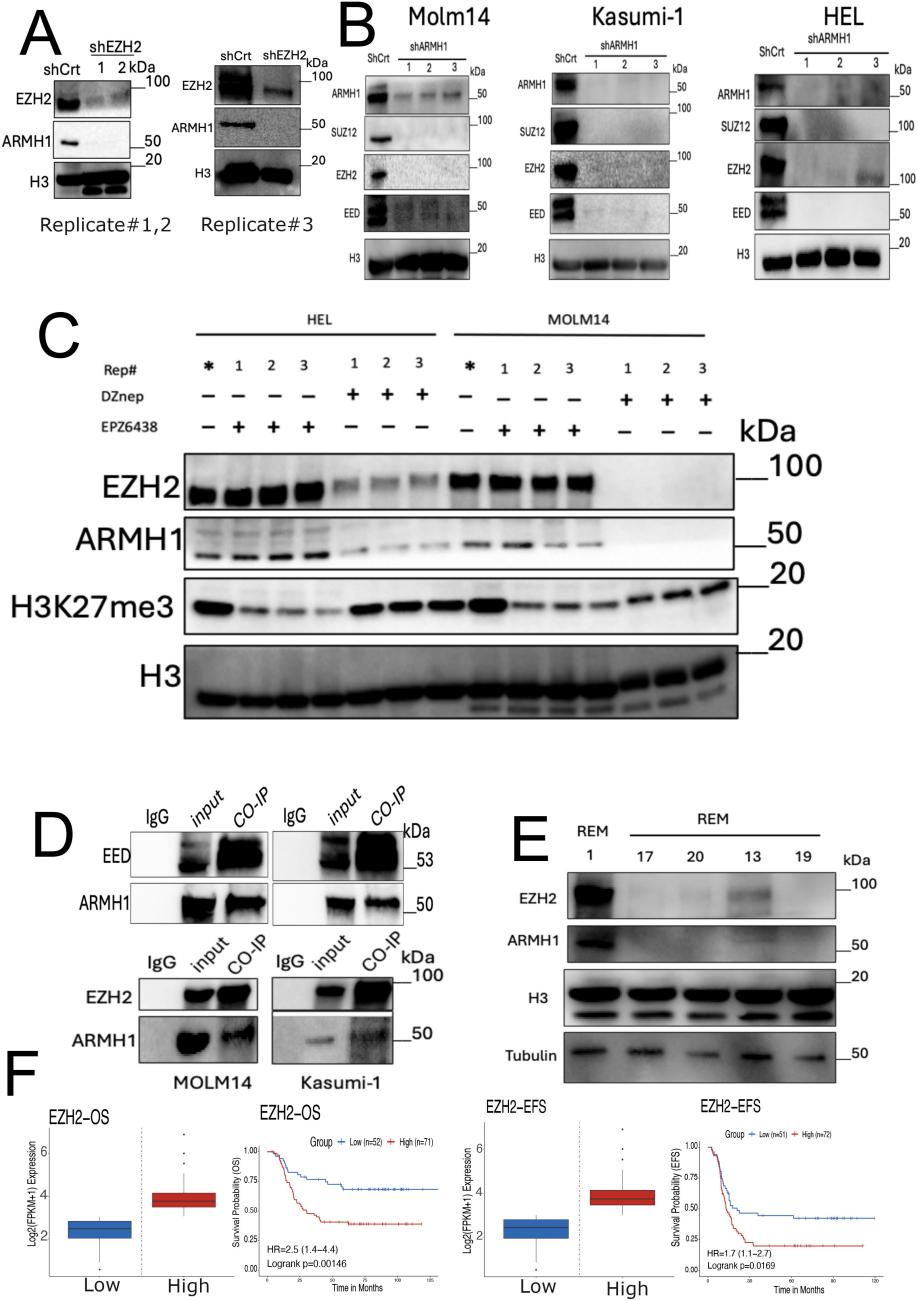
*ARMH1* is a novel interactant of *EZH2* in leukemia. **(A)** Western blot of MOLM14 cell lysates probed with *ARMH1* and *EZH2* antibody and total H3 antibody as loading control with lentiviral transduced *shEZH2* or shCrt (3 independent replicates). Left panel: replicates#1,2 of *shEZH2* (the lower bands in H3 are non-specific bands), and right panel: *shEZH2* replicate#3. **(B)** Western blot of cell lysates of MOLM14, Kasumi-1, and HEL, probed with *ARMH1*, *EED, EZH2*, and *SUZ12* antibody and total H3 as loading control with lentiviral transduced *shARMH1* or shCrt (3 independent replicates). **(C)** Western blot of detecting *ARMH1*, *EZH2*, and H3K27me3 in MOLM14 and HEL92.1.7 cell lines in (3 independent replicates) after treatment of EPZ6438 (150uM) and DZNep (0.5uM) over 72 hours. The membrane was first probed with H3K27me3 and then stripped and probed with total H3. (Lower bands in H3 are non-specific bands). **(D)** Western blot on Co-IP pull-down using *EED (Upper Panel) and EZH2 (Lower Panel)* antibodies for MOLM14 and Kasumi-1 cells extract. The membranes were probed with *EZH2, EED*, and *ARMH1* antibodies. **(E)** Western blot on patient samples collected at disease diagnosis from patients with future relapse (patient#1) and remission (patients#13,17,19,20) probed with *ARMH1*, *EZH2*, and total H3 and Tubulin as loading control. **(F)** Kaplan-Meier curve shows overall survival (left), and event-free survival (EFS) (right) based on the expression of *EZH2*. The Survival Genie platform (https://bhasinlab.bmi.emory.edu/SurvivalGenie2/home) was used to perform survival analysis. The samples were grouped into “low” and “high” groups based on an optimal threshold calculated with the cutP method. Using these groupings, we calculate the survival probability and generate Kaplan Meier curves with corresponding log-rank p-value, which represents the significance of the difference between the curves for “high” and “low” expression groups. A Cox proportional hazard ratio (HR) is calculated between the high and low samples, an HR>1 represents poor survival, whereas an HR<1 represents an association with better survival. The asterisk (*) means control for replicates.

Studies have previously demonstrated that the enhancer of zeste homolog 2 (*EZH2*) plays a regulatory role in Lipid Metabolism, and simultaneous targeting of *EZH2* and Fatty Acid Synthesis results in cancer cell death (46). Previous studies have shown that *EZH2* increases lipid synthesis in a variety of cancers (47–49) and is associated with poor prognosis (50, 51). Moreover, *EZH2* has been reported as a multifaced regulator across different types of cancers, potentially functioning both as an oncogene and a tumor-suppressor gene in cancer (52, 53). However, recent studies have underscored the remarkable role of *EZH2* in hematologic malignancies and leukemogenesis (54, 55). Our data suggest that *ARMH1* may serve as an interactant of *EZH2* associated with high-risk and therapeutic resistance patients in pediatric AML. We observed that the knockdown of either *EZH2* or *ARMH1* in MOLM14 cells diminishes the expression of both *EZH2* and *ARMH1* proteins (**Figure 5A**). Additionally, in *ARMH1* knockdown cells, we observed downregulation of Embryonic Ectoderm Development (*EED*) and Suppressor of Zest 12 (*SUZ12*) the core component of the Polycomb repressive complex 2 (PRC2) along with *EZH2*, which highlights *ARMH1’s* role in PRC2 complex destabilization (**Figure 5B**). Similar results were observed in the HEL92.1.7 cell line for *EZH2* knockdown (**Supplementary Figure 2F**). To further support the possibility of *EZH2* and *ARMH1* physical interaction, we either inhibited the enzymatic activity of *EZH2* using EPZ6438 (an inhibitor targeting the zest domain of *EZH2*) or destabilized *EZH2* protein and PRC2 complex using DZNep (3-Deazaneplanocin A). After treatment of AML cells with EPZ6438, while protein level *EZH2* was not altered, its enzymatic activity was downregulated as determined based on reduced levels of H3k27me3 (**Figure 5C**). We observed that the expression of *ARMH1* remained at almost the same level or slightly changes. On the other hand, after DZNep treatment, a potent destabilizer of the *EZH2*, the *ARMH1* protein expression was decreased significantly along with a decrease in levels of *EZH2* (**Figure 5C**). This data indicates that *ARMH1* and *EZH2* may have physical interaction together, which occurs post-translationally. For further investigation of *EZH2* and *ARMH1* interaction, we performed Co-Immunoprecipitation (CO-IP) by pulling down *EZH2* and *EED* in MOLM14 and Kasumi-1 cell lines. The pull-down product analysis using western blot showed a signal for both *EZH2, EED*, and *ARMH1* proteins, suggesting a possible physical interaction among these proteins (**Figure 5D**). Also, the protein expression analysis of *EZH2* and *ARMH1* in patient samples has shown a higher expression for both in diagnostic samples from patients who experienced relapse (patient#1) as compared to those who achieved remission (patients#13,17,19,20) (**Figure 5E**). Moreover, the survival analysis on *EZH2* expression in the TARGET AML dataset depicted a significant association between high expression of *EZH2* and poor overall survival (*P=0.00249*), and event-free survival (*P=0.033*) (**Figure 5F**). Taken together, this highlights the importance of *ARMH1* as *EZH2* interactant at the post-translational level and provides insights into its involvement in high-risk factors and therapeutic resistance mechanisms, in pediatric AML.

## Discussion

The emergence of resistance to conventional chemotherapies is increasing, and it can be stimulated by different mechanisms and finally leads to adverse outcomes in pAML patients, while cytogenetic assessments and molecular abnormalities provide pivotal prognostic indicators and predictive capabilities, pAML continues to exhibit high mortality rate due to lack of robust biomarker that reliably indicates a relapse (56, 57). In the current study, we report that *ARMH1* may serve as a potential biomarker for relapse in pediatric AML. Recently, *ARMH1* has been identified as a potential biomarker gene for the immunological score-based risk stratification signature in oral squamous cell carcinoma (58), but *ARMH1* is still reported to be an unknown function gene and has not been studied in detail in cancer, especially leukemias. This is the first study attempt to characterize *ARMH1* at both functional and molecular levels in pediatric AML. Our data suggests that the knockdown of *ARMH1* leads to a significant reduction in cell proliferation and migration as well as slightly enhances response toward conventional chemotherapy. Conversely, overexpression of *ARMH1* leads to increasing cell proliferation and migration rates. Interestingly, these effects are similar to very well-studied genes that play a critical role in tumorigenesis in AML, such as *c-Kit* proto-oncogene, Runt-related transcription factors, *RUNX1*, and *FLT3* (59–61). In the current research, we observed that *ARMH1* has a notable role in the progression of AML, correlating significantly with relapse and poor clinical outcomes. Results from another team reveal that the mitochondrial fatty acid synthesis pathway assumes a critical role in the progression of AML (62, 63). Furthermore, inhibition of the mitochondrial fatty acid synthesis pathway in combination with conventional chemotherapy protocol has shown a notable impact on inhibiting leukemia progression (64). Findings from other teams signify that AML exhibits a dependency on mitochondrial metabolism and mitochondrial fatty acid synthesis pathways (65). In this study, to characterize the molecular landscape of *ARMH1* perturbation effects, we leveraged RNA-seq and Pathway analysis on *ARMH1* knockout, overexpressing, and control cells. The RNA-seq analysis enrichment of the mitochondrial fatty acid synthesis pathway in the genes that are consistently positively correlated with *ARMH1* expression across cell lines. Our Seahorse assay data also demonstrated that knockdown of *ARMH1* leads to a significant reduction in mitochondrial OCR. This finding highlighted the potential linkage of *ARMH1* to the mitochondrial fatty acid synthesis pathway in AML tumorigenesis. Additionally, our data indicated that *ARMH1* has a role in the regulation of the cell cycle. *CDCA7* has been consistently reported in many research studies to play a remarkable role in tumor progression in various types of cancers (38, 66–68). In the current paper, an analysis of differential gene expression using bulk RNA seq data has shown that *CDCA7* ranks as the gene showing significant co-expression with *ARMH1*, during *in vitro* studies, *ARMH1* knockdown leads to downregulation of *CDCA7*, and knockdown *CDCA7* causes downregulation of *ARMH1*. This finding highlighted the regulatory role of *ARMH1* in influencing the expression of *CDCA7* in pediatric leukemia, which has never been reported. Also, our data indicated that the knockdown of *ARMH1* leads to the upregulating of the checkpoint inhibitor gene, *P21*, resulting in cell cycle arrest. In the current study, we also identified that the knockdown of *ARMH1* leads to the destabilization of the PRC2 complex and the degradation of *EZH2* and *EED*, a core component of the PRC2 complex. Our data highlighted that *ARMH1* and *EZH2* may have protein-protein interactions, indicating functional interdependence between these two genes. Several studies have reported that *EZH2* downregulation has been associated with poor prognosis (Stomper et al., 2021; Kempf et al., 2021; Göllner et al., 2017). On the other hand, multiple studies across solid tumors show contrary results, while *EZH2* overexpression has been linked to poor prognosis (69–71). These studies highlight a dual and puzzling role of *EZH2* in cancer progression that might be influenced by complex cancer genetics and microenvironment. Our finding suggests that in pediatric AML the downregulation of ARMH*1* along with *EZH2* has been associated with good outcomes. Taken together, the absence of *ARMH1* is a good prognostic marker as its upregulation is associated with aggressive disease, so it can be used in addition to cytogenetics for identifying patients with high relapse probability. It is involved in two cancer hallmark pathways related to metabolism and cell cycle, making it a good therapeutic target. Fatty acid metabolism-based targeting might provide a novel way to treat high-risk pediatric AML.

## Conclusion

In conclusion, our study identified *ARMH1* as a novel biomarker for aggressive pediatric AML, demonstrating *ARMH1’s* significant association with disease progression and relapse. Through comprehensive analysis, we elucidated the impact of *ARMH1* on leukemic cell proliferation, migration, and chemotherapy sensitivity, as well as *its* involvement in mitochondrial fatty acid synthesis and cell cycle regulation. These findings underscore *ARMH1 as a* potential prognostic indicator for patient stratification and a therapeutic target for enhancing clinical remission rates. Further investigation into *ARMH1’s* role in AML pathogenesis will be required to improve advancing personalized treatment strategies and improving pAML patient outcomes.

### Limitations

One limitation of this study is the lack of animal models to complement the findings obtained from *in vitro* studies. Consequently, the conclusion of findings solely from *in vitro* or clinical studies may not capture the entirety of the pathobiological role of *ARMH1* in pediatric leukemia. Limited understanding of the *ARMH1* effect on immune cells is another limitation of the study.

## Methods

### Single-cell transcriptome analysis samples, assay, and sequencing

The single-cell AML dataset was generated in our lab previously, and *ARMH1* was identified among a 7-gene signature that distinguished AML malignant cells (14). Viable frozen AML bone marrow samples were obtained from the Aflac Cancer and Blood Disorders Center, Children’s Healthcare of Atlanta, and processed for generating high-quality single-cell RNA sequencing data on 20 samples. These BM samples were viably thawed and processed for single-cell transcriptome profiling using the 10x Chromium Controller, followed by library preparation with 10x Genomics Single Cell 3`v3, and 5`v1 reagent kits. Sequencing was performed on the Novaseq 6000 platform. The raw scRNA-seq data was demultiplexed, filtered, and aligned to the human reference genome (hg38) using Cell Ranger software by 10x Genomics (v3). This output single-cell count matrices for each sample, which were then pre-processed, integrated, and normalized using *Seurat* package (v4.3) functions (72). To account for the two batches of data generated from our lab (batch 1: samples 1-15, batch 2: samples 16-20), the integration anchors method (*Seurat*) was used to integrate and perform batch correction. Log normalization, scaling, PCA, UMAP, and clustering were applied to generate processed single-cell expression data for downstream analysis. Among the 20 patients in our dataset, seven experienced relapse, and 13 experienced clinical remission. This dataset is publicly available on the Gene Expression Omnibus repository under accession GSE235923. In addition to our lab’s 20-sample AML dataset, we also included a publicly available 8-sample AML scRNA-seq dataset from the GEO repository (GSE154109) (12). *Seurat* functions were again used for log normalization, scaling, PCA, UMAP, and clustering to generate normalized single-cell expression data for downstream analysis. Among the patients from this dataset (12), six experienced remission, and two experienced relapse.

### Bulk RNA sequencing analysis on cell lines after perturbing *ARMH1* levels

Total RNA was extracted from cell lines of different groups (HEL shCrt/EV (n=3), HEL *shARMH1* (n=3), HEL *ARMH1* Oe (n=2), MOLM14 shCrt/EV (n=3), MOLM14 *shARMH1* (n=2) and MOLM14 *ARMH1* Oe (n=2) using RNeasy Micro Kit (Cat# 74004, Qiagen) according to the instructions provided by the manufacturer. RNA concentration was evaluated by photometric measurement at 260/280 nm, and quality was assessed using the Agilent Bioanalyzer platform. For each group, sequencing was performed on two or three biological replicates. Double-stranded cDNA sequencing libraries were generated using the Illumina TruSeq kit per the manufacturer’s protocol. High-quality libraries were sequenced on an Illumina Novaseq 6000 platform. To achieve comprehensive coverage for each sample, we generated ~20–30 million paired-end reads.

The raw sequencing data was processed to remove any adaptor, PCR primers, and low-quality transcripts using FASTQC (73) and fastx. These provide a very comprehensive estimate of sample quality based on read quality, read length, GC content, sequence duplication, adaptor, and PCR primer contamination. These high-quality, clean reads were aligned against the human genome using the hisat2 aligner (74). We used GRCh38 human genome assembly as the reference genome for alignment. Gene expression measurement was performed from aligned reads by counting the unique reads using the htseq-count algorithm (75). The read count-based gene expression data was normalized based on library complexity and gene variation using the Voom algorithm (76). The normalized count data underwent comparison among groups using the limma method (77) to identify differentially expressed genes (raw *p < 0.05*). Principal Component Analysis (PCA) was used to capture the variation in the data across different biological groups. Supervised analyses focused on identifying differentially expressed genes through four distinct comparisons: overexpression vs. control and knockdown vs. control for each cell line. Our primary aim was to identify co-expressing genes that are upregulated and downregulated in *ARMH1* Oe and *shARMH1* cell lines respectively, but we were also interested in identifying counter-expressing genes that are downregulated in *ARMH1* Oe and upregulated in *shARMH1*. Genes were selected for downstream analysis based on their consistency in differential expression patterns and statistical significance across the different contrasts. Due to limited overlap between the four groups, genes were included if they exhibited expression changes consistent with the direction of interest (logFC > 0 in overexpression and logFC < 0 in knockdown for co-expressed genes and the opposite for counter-expressed genes) and demonstrated statistical significance (*P < 0.05*) in at least three out of the four contrasts.

Pathway enrichment analysis was conducted using the Enrichr tool (77) to identify pathways significantly affected by *ARMH1* perturbations. Specifically, enrichment analysis was carried out separately for *ARMH1* co-expressed and counter-regulated genes utilizing the ‘WikiPathway 2023 Human database. Pathways with a *P-value < 0.05* were determined to be significantly perturbed by *ARMH1*. To capture subtle pathway level perturbations due to *ARMH1*, Gene Set Variation Analysis (GSVA) was performed using the gsva R package (78). Using the Hallmark, C2: CP, C3: TFT, and C5: GO gene sets from MSigDB (79) as reference, the voom-derived normalized expression values were transformed through GSVA into sample-wise enrichment scores for each gene set. Subsequently, the limma package (77) was utilized to conduct differential testing on these enrichment scores obtained from GSVA. This step enabled the identification of gene sets that are significantly (*P < 0.05*) upregulated or downregulated in response to *ARMH1* overexpression or knockdown, respectively, or vice versa (78–80). Next, the BioNERO package (81) was utilized to construct gene co-expression networks from genes that were co-expressed with *ARMH1*. Using the voom-derived normalized expression values, we constructed signed hybrid co-expression networks for each cell line employing Pearson’s correlation metric with a module merging threshold of 1.0. Hub genes within the resulting network were identified based on BioNERO’s principles of module membership and degree of nodes. To mitigate potential unwanted effects from genes with low log fold-change values, this process was iterated by iteratively removing the gene with the lowest average logFC among the four contrasts and re-running the network analysis with the same parameters as mentioned above. Hub genes that were common between the MOLM14 and HEL cell lines in any of these runs were selected for further investigation. Finally, we utilized Survival Genie (19) to conduct survival analysis on genes significantly (*P < 0.05*) upregulated in *ARMH1* Oe and downregulated in *shARMH1* in at least three contrasts. This analysis aimed to investigate whether disparities in gene expression levels correlated with survival probabilities in the TARGET-AML patient cohort. Employing the cutp method from the R package survMisc (https://cran.r-project.org/package=survMisc), we partitioned the TARGET-AML cohort into two distinct groups, characterized by relatively high or low expression levels of the *ARMH1*-perturbed genes. Subsequently, Kaplan-Meier survival curves were constructed, and a hazard ratio, indicative of the magnitude of differences between these high and low expression groups, was computed. The confidence interval and log-rank p-value for the hazard ratio were then determined to assess the significance of any observed differences. Additionally, we further utilized the TARGET-AML cohort to examine the association of *ARMH1* expression with risk in AML. Primary BM samples from TARGET-AML were grouped into high-risk – MLL cytogenetic subtype – and standard risk – *RUNX1, inv (16)* cytogenetic subtypes. Then, voom-derived normalized expression values were obtained for *ARMH1*, and expression was compared in high-risk and standard-risk samples using a Wilcoxon rank-sum test.

### Cell culture methods

Leukemia cells MOLM14, HEL92.1.7, Kasumi-1, and normal cell HEK293T were generously provided by Dr. Douglas K. Graham and Dr. Deborah DeRyckere’s labs (Emory University, Department of Pediatric, Hematology, and Oncology division) and Mesenchymal Stromal Cells (MSC) were kindly provided by Dr. Subra Kugathasan’s lab (Emory University, Pediatric Department, Children’s Healthcare of Atlanta Combined Center for Pediatric Inflammatory Bowel Disease). Leukemia cells were cultured in RPMI 1640 media supplemented with 10% FBS, 100U/ml penicillin/streptomycin, and 1% Glutomax. HEK293T and MSC cells were cultured in DMEM F12 media supplemented with 10% FBS 100U/ml penicillin/streptomycin and 1% Glutomax. All cell lines were checked periodically for Mycoplasma contamination using MycoAlert^®^ Mycoplasma Detection Kit (Cat#LT07-418). During the *in vitro* experiments, the cells were free of Mycoplasma contamination.

### Cell viability detection

To detect cell viability, we used Bio-Rad Trypan Blue (Cat#1450021) and automated cell counter TC20 (Cat#1450102). The cells with more than 90% viability were utilized for experiments.

### Lentivirus packaging and transduction

To generate *ARMH1*, *EZH2*, and *CDCA7* knockdown cells as well as *ARMH1* overexpression cells, the lentivirus was generated with transfection of shRNAs (pLKO.1) and the Lentiviral Packaging Kit (Lenti-vpak, Cat#TR30037, OriGene) by following manufacturer protocol. Three shRNAs were used for *ARMH1* knockdown, with clone IDs as follows: TRCN0000168648 with target sequence: GCTGAGGACTTGTACATGAAA in 3UTR, TRCN0000172632 with target sequence: GAGTCACATCCTCGACAAGTT in CDS, and TRCN0000336686 with target sequence: CAAGTGTATAAAGGTCTAATA in CDS. One validated shRNA was used for *EZH2* knockdown; the clone ID was TRCN0000040077 with the target sequence: CCCAACATAGATGGACCAAAT in CDS. One validated shRNA was used for *CDCA7* knockdown; the clone ID was TRCN0000017937 with target sequence: CCAGTTATTGGCGGAATTGAA in CDS. We utilized *ARMH1* cloned in the Open Reading Frame plasmid (ORF_pLX-317) for overexpression of *ARMH1*. All plasmids were purchased from Millipore Sigma, Inc. Plasmid extraction: Bacteria derived from each glycerol stock of shRNA and ORF clones were distributed onto agar-ampicillin-positive plates, following the incubation of 18 hours in a 37°C dry incubator, a single colony was picked and grown in LB broth at 37°C and 200 rpm for 18 hours in a shaker incubator. Subsequently, the plasmids were extracted from bacteria utilizing PureLink™ HiPure Plasmid Midiprep Kit (Cat#K210004). Lentivirus particles and transduction to target cells: After reaching HEK293T cell confluency to 80% in 6 wells plate, the HEK293T cells were incubated with the lentivirus packaging plasmids and shRNA/ORF plasmids for 24 hours following the manufacturer’s protocol. After 24 hours, the media was changed to fresh media (complete RPMI 1% penicillin/streptomycin, 10% FBS and 1% Glutomax). During the 72 hours, lentiviruses were collected, filtered through a 0.45um filter, and stored at -80°C. The target leukemic cells were cultured in the presence of polybrene (8 ug/ml) and lentivirus for 24 hours; then, the targeted cells were washed with 1X PBS and grown for an additional 2 days. The transduced cells were selected with puromycin dihydrochloride (2 ug/ml) (Cat#A1113803, ThermoFisher) in two weeks. All experiments were conducted as three independent biological replicates.

### Co-immunoprecipitation

On day 1, the total protein was extracted from the cell lines and protein concentration was measured with BCA assay (Cat#A55860, ThermoFisher). Separately, the 4ug of *EZH2* antibody (Cat#A16846, ABclonal) was mixed with 50ul of protein G (Cat#16-266EDM Millipore). Increased volume to 500ul by adding 1X PBS and incubated overnight at 4C. On day 2, the lysate was prepared by mixing 1ml of 1X PBS with 50ul of protein G, which was pre-cleared, then centrifuged at 5000rpm for 2 minutes at 4C. The supernatant was aspirated, resuspended in 500ul of RIPA buffer, and centrifuged at 5000 rpm for 2 minutes at 4C. Then, the supernatant was collected, the protein G and antibody mixture were added, and incubated overnight at 4C. After overnight incubation on day 3, the lysate was centrifuged at 5000 rpm for 2 minutes at 4C, washed twice with cold PBS, resuspended in western blot elution buffer, and ran on SDS gel.

### Western blot

Cells were lysed in 1X RIPA Lysis Buffer (Cat#89900, ThermoFisher) with 1X Halt™ Protease Inhibitor Cocktail, EDTA-free (100X) (Cat#78425, ThermoFisher) for 30 min on ice. Protein concentration was measured by Pierce™ Dilution-Free™ Rapid Gold BCA Protein Assay (Cat#A55860, ThermoFisher). Protein extracts were resolved by SDS–PAGE on 4–20% Mini-PROTEAN^®^ TGX™ Precast Protein Gels (Cat# 4561093, Bio-Rad). Post SDS-PAGE, the gels were blotted onto the membrane, 0.2 µm (Cat#1620112, Bio-Rad), and probed with the following antibodies: *ARMH1* (Cat #PA5-71024, ThermoFisher), β-Actin mouse mAb (Cat #3700, Cell signaling), H3 (Cat#A2348, ABclonal), *SUZ12* (A7786, AB clonal), *EED* (A5371, AB clonal) H3K27me3 (Cat#A2363, ABclonal), *EZH2* (Cat#A16846, ABclonal), *CPT1A* (Cat#A5307, AB clonal), *CDCA7* (Cat#A15472, ABclonal), GAPDH (Cat#14C10, cell signaling). Secondary antibodies used: anti-mouse IgG, HRP-linked Antibody (Cat#7076, Cell signaling) and Anti-rabbit IgG, HRP-linked Antibody (Cat# 7074, Cell signaling), TidyBlot secondary antibody (Cat#STAR209p, Bio-Rad). The signal was developed with SuperSignal™ West Pico PLUS Chemiluminescent Substrate (Cat#PI34578, FisherScentific), and images were detected with ChemiDoc MP instrument (Cat#12003154, Bio-Rad). We used TBST (Cat#40120065-2, BioWorld) for washing membranes. Primary antibody was diluted in 1X TBST/5%BSA, and secondary antibodies were diluted in 1X TBST/5% dry milk.

### Cell proliferation assay

A total of 2500 cells with viability > 90% were cultured in 96-well cell culture plates in a total volume of 100ul with complete cell culture media. Following an incubation period of 72 hours (37°C, 5%CO2), 10ul of CCK-8 kit (Dojindo Cat#CK04-01) solution was added to each well. This was followed by further incubation for 3 hours at 37°C, 5% CO2, and the absorbance was measured at 450 nm in plate reader (BMG, Labteck, CLARIO Star).

### Cell migration assay

The CytoSelectTM 24-Well Cell Migration Assay (3μm, Fluorometric Format; Cat# CBA-103, Cell Biolabs, Inc) was used for cell migration assay. We followed the manufacturer’s protocol, and the Relative Florescent Unit (RFU) was measured using a fluorescence plate reader (BMG, Labteck, CLARIO Star).

### Dose-response assay

Cells were cultured at 2500 count per well with viability > 90% in a total volume of 50ul cell culture complete media. On the day of treatment, Cytarabine USP grade (Cat#1162002; Sigma Aldrich) was added to the medium at various concentrations (Control wells are 0 nanomolar (nM) concentration while Cytarabine treatment concentrations were 25,50,75,100,125,150,175,250,300 nM). The reaction was terminated after 72 hours by adding 100 μl/well of CellTiter-Glo^®^ 2.0 (Cat#G9241, Promega), and cell viability was assessed by measuring the absorbance at 490nm in a 96-well plate reader (BMG, Labteck, CLARIO Star). The doses that decrease cell viability to 50% (IC_50_) were analyzed using a nonlinear regression log (inhibitor) vs. normalized response (three parameters) with GraphPad Prism (version 10) software.

### Seahorse and OCR measurement

We utilized the Seahorse XFe24 Analyzer (Agilent) to measure the OCR of live cells with more than 90% viability. A day before the assay: Turned on the Agilent Seahorse XFe/XF analyzer for warm-up overnight (minimum of five hours). Hydrate a sensor cartridge in Seahorse XF (Cat#102342-100, Agilent) calibrant at 37°C in a non-CO2 incubator overnight. On the day of assay, the first assay medium was made: Glucose (Cat#G7021-100mg, Sigma) 11mM, Sodium Pyruvate (Cat#SH3023901, Hyclone) 1mM, Glutamine (Cat#17-605E, Lonza) 2mM and XF Base medium (Cat#102353-100, Agilent) in volume of 97ml. After preparing the media,1N NaOH (Cat#3722-11, Baker) was prepared to adjust the pH of the medium to 7.4. Drug preparation: Oligomycin (Cat#495455, Sigma) 2.5uM, FCCP (Cat#C2920, Sigma) 0.5uM, Antimycin (Cat#A8674, Sigma), Rotenone (Cat#R8875, Sigma) each drug was 2uM. The Cell-Tak coated XF24 cell culture was prepared, and the assay was set up as described in the manufacturer’s protocol.

### Data analysis and statistics

Statistical analysis of data was performed with GraphPad Prism 10 software (GraphPad Software Inc., La Jolla, CA) or with statistical functions developed using R language. All data are presented as the means ± SD or SEM. Significant differences were determined using a two-tailed student’s *t*-test, ANOVA test, or Wilcoxon rank-sum test.

## Data Availability

The datasets presented in this study can be found in online repositories. The names of the repository/repositories and accession number(s) can be found in the article/**Supplementary Material**.
