# Supplementary material for: ARMH1 is a novel marker associated with poor pediatric AML outcomes that affect the fatty acid synthesis and cell cycle pathways
Source: Front Oncol. 2024 Dec 5;14:1445173. doi: 10.3389/fonc.2024.1445173 (PMC11655347; doi:10.3389/fonc.2024.1445173)
Supplement: Supplementary file 1 [file DataSheet1.pdf]

Supplementary Materials for

***ARMH1 is* a novel marker associated with poor pediatric AML outcomes that affect the fatty acid synthesis and cell cycle pathways.**

Mojtaba Bakhtiari[1,3], Sean Jordan[1,3], Hope L. Mumme[1,3], Richa Sharma[4], Mala Shanmugam[4], Swati S. Bhasin[1, 2] Manoj Bhasin[1,2,3, #]

1. Aflac Cancer and Blood Disorders Center, Children Healthcare of Atlanta, Atlanta, GA
2. Department of Pediatrics, Emory University, Atlanta, GA
3. Department of Biomedical Informatics, Emory University, Atlanta, GA
4. Department of Hematology and Medical Oncology, Emory University School of Medicine, Atlanta, GA

#Corresponding author

Manoj K. Bhasin, MS, PhD

Aflac Cancer and Blood Disorders Center

Children Healthcare of Atlanta

Health Sciences Research Building II

1750 Haygood Dr NE, N320

Emory School of Medicine

Atlanta, GA 30322.

E-mail: manoj.bhasin@emory.edu

Telephone: (404) 712-9849

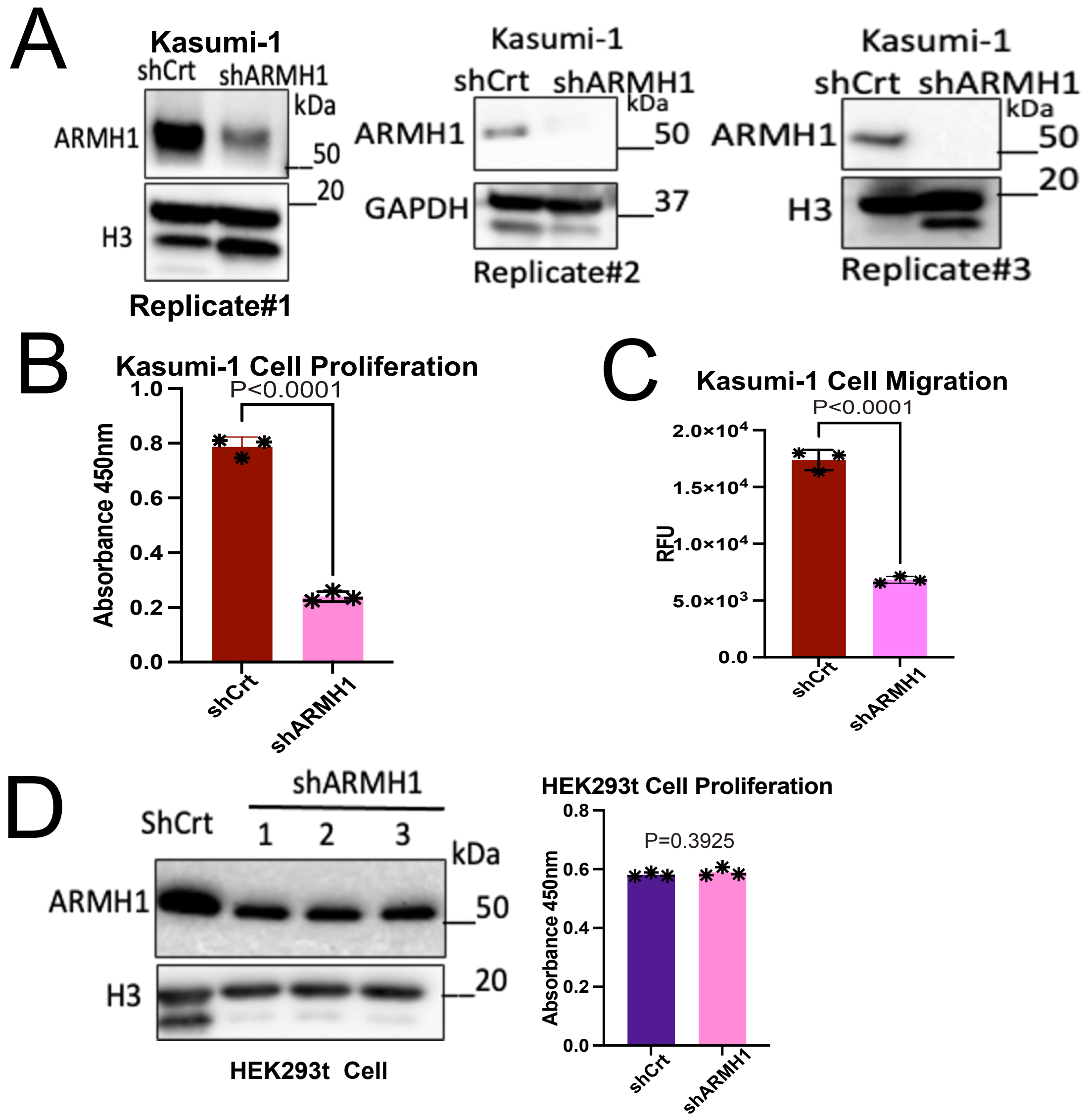
A
Kasumi-1
shCrt
shARMH1
kDa
ARMH1
50
20
H3
Replicate#1
GAPDH
37
Replicate#2
Replicate#3
B
Kasumi-1 Cell Proliferation
P<0.0001
Absorbance 450nm
1.0
0.8
0.6
0.4
0.2
0.0
C
Kasumi-1 Cell Migration
P<0.0001
RFU
2.0×10⁴
1.5×10⁴
1.0×10⁴
5.0×10³
D
ShCrt
1
2
3
HEK293t Cell
HEK293t Cell Proliferation
P=0.3925

Fig. S1. ***ARMH1*** **knockdown decreased cell proliferation, migration and increased sensitivity to chemotherapy**

(A) Western blot of Kasumi-1 cells lysates probed with *ARMH1* antibody and total H3, GAPDH as loading control with lentiviral transduced *shARMH1* and shCrt (3 independent replicates). Left panel: replicate#1 of *shARMH1*(Lower bands in H3 are non-specific bands), middle panel: replicate#2 of *shARMH1* (Lower bands in GAPDH are non-specific bands), and right panel: replicate#3 of *shARMH1* (Lower bands in H3 are non-specific bands). **(B)** Bar plot showing cell proliferation measured by CCK-8 in Kasumi-1 *shARMH1* cells along with shCrt. Statistical analysis: Unpaired student's t-test (n=3), data are means (absorbance in 450nm) +/-SEM of independent biological replicates. **(C)** Bar plot showing the migration assay was conducted by using the fluorometric format in Kasumi-1 cell line with *ARMH1 shARMH1* along shCrt. Statistical analysis: Unpaired student's t-test (n=3), data are means of Relative Florescence Unit (RFU) +/-SEM of independent biological replicates. **(D)** Left panel: Western blot of HEK293T cell lysates probed with *ARMH1* antibody and total H3 antibody as loading control with lentiviral transduced *shARMH1* or shCrt (3 independent replicates) (Lower bands in H3 are non-specific bands). Right panel: Bar plot showing cell proliferation measured by CCK-8 in HEK293T in *shARMH1* cells along with shCrt. Statistical analysis: Unpaired student's t-test (n=3), data are means (absorbance in 450nm) +/-SEM of independent biological replicates.

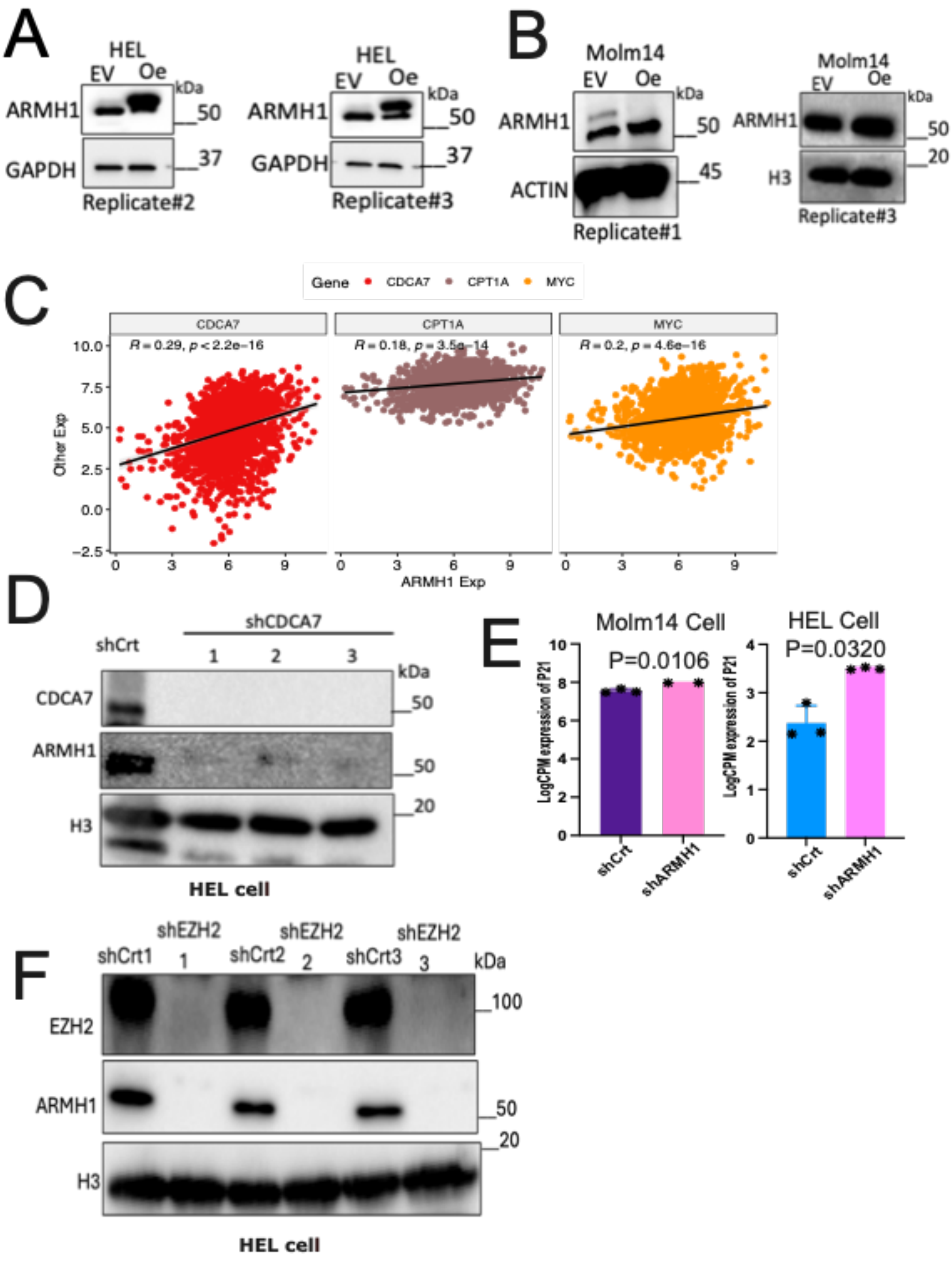
A
HEL
EV Oe
kDa
ARMH1
50
GAPDH
37
Replicate#2
HEL
EV Oe
kDa
ARMH1
50
GAPDH
37
Replicate#3
B
Molm14
EV Oe
kDa
ARMH1
50
ACTIN
45
Replicate#1
Molm14
EV Oe
kDa
ARMH1
50
H3
20
Replicate#3
C
Gene CDCA7 CPT1A MYC
CDCA7
R = 0.29, p < 2.2e−16
CPT1A
R = 0.18, p = 3.5e−14
MYC
R = 0.2, p = 4.6e−16
Other Exp
ARMH1 Exp
D
shCrt
shCDCA7
1 2 3
kDa
CDCA7
50
ARMH1
50
H3
20
HEL cell
E
Molm14 Cell
P=0.0106
LogCPM expression of P21
shCrt
shARMH1
HEL Cell
P=0.0320
LogCPM expression of P21
shCrt
shARMH1
F
shCrt1 shEZH2 1 shCrt2 shEZH2 2 shCrt3 shEZH2 3
kDa
EZH2
100
ARMH1
50
H3
20
HEL cell

Fig. S2. Showing *ARMH1* overexpression in western blot, *ARMH1* co-expression with cell cycle regulators: *CDCA7, EZH2*, *c-MYC* and *P21*

(A) Western blot of HEL92.1.7 cell lysates probed with *ARMH1* antibody and GAPDH antibody as loading control with lentiviral transduced ORF (pLX-317) or EV. Left panel: replicate#2 of HEL92.1.7 *ARMH1* Oe, right panel: replicate#3 of HEL92.1.7 *ARMH1* Oe. **(B)** Western blot of MOLM14 cell lysates probed with *ARMH1* antibody and ACTIN antibody as loading control with lentiviral transduced ORF (pLX-317) or EV. Left panel: replicate#1 of MOLM14 *ARMH1* Oe, right panel: replicate#3 of MOLM14 *ARMH1* Oe. **(C)** Utilizing the TARGET-AML Bulk RNA-seq dataset, a scatter plot of normalized *ARMH1* expression on x-axis and normalized expression of genes of interest on y-axis. A Pearson correlation test was performed to assess the relationship between *ARMH1* and *CDCA7*, *CPT1A*, and *MYC* expression. Corresponding Pearson correlation coefficients (R values) are reported on the plots, along with p-values. **(D)** Western blot of HEL92.1.7 cell lysate probed with *ARMH1*, *CDCA7,* and total H3 as loading control with lentiviral transduced *shCDCA7* or shCrt. (3 independent replicates). Lower bands in H3 are non-specific bands. **(E)** Bar plots of expression of *P21* in *shARMH1* and shCrt in MOLM14 cell line (Left) and HEL92.1.7 (Right). Statistical analysis: unpaired student's t-test (n=3), data are mean of log CPM expression+/-SEM of *P21* from independent biological replicates. **(F)** Western blot of HEL92.1.7 cell lysates probed with *ARMH1* antibody and total H3 antibody as loading control with lentiviral transduced *shEZH2* or shCrt. (3 independent replicates). Lower bands in H3 are non-specific bands.
